# Identification of drivers of Rift Valley fever after the 2013–14 outbreak in Senegal using serological data in small ruminants

**DOI:** 10.1371/journal.pntd.0010024

**Published:** 2022-02-02

**Authors:** Ismaila Seck, Modou Moustapha Lo, Assane Gueye Fall, Mariane Diop, Mamadou Ciss, Catherine Béatrice Cêtre-Sossah, Coumba Faye, Mbargou Lo, Adji Mareme Gaye, Caroline Coste, Cécile Squarzoni-Diaw, Rianatou Bada Alambedji, Baba Sall, Andrea Apolloni, Renaud Lancelot

**Affiliations:** 1 Food and Agriculture Organization of the United Nations (FAO), Regional Office for Africa (RAF), Accra, Ghana; 2 Direction des Services vétérinaires (DSV), Dakar, Sénégal; 3 Institut Sénégalais de Recherches Agricoles (ISRA), Laboratoire National de l’Élevage et de Recherches Vétérinaires (LNERV), Dakar-Hann, Sénégal; 4 ASTRE, Univ. Montpellier, CIRAD, INRAE, Montpellier, France; 5 Centre de cooperation internationale en recherche agronomique pour le développement (CIRAD), UMR ASTRESainte Clotilde, la Réunion, France; 6 Centre de cooperation internationale en recherche agronomique pour le développement (CIRAD), UMR ASTRE, Montpellier, France; 7 École Inter- États des Sciences et Médecine Vétérinaires de Dakar, Dakar, Sénégal; University of Iowa, UNITED STATES

## Abstract

Rift Valley fever (RVF) is a mosquito-borne disease mostly affecting wild and domestic ruminants. It is widespread in Africa, with spillovers in the Arab Peninsula and the southwestern Indian Ocean. Although RVF has been circulating in West Africa for more than 30 years, its epidemiology is still not clearly understood. In 2013, an RVF outbreak hit Senegal in new areas that weren’t ever affected before. To assess the extent of the spread of RVF virus, a national serological survey was implemented in young small ruminants (6–18 months old), between November 2014 and January 2015 (after the rainy season) in 139 villages. Additionally, the drivers of this spread were identified. For this purpose, we used a beta-binomial (BB) logistic regression model. An Integrated Nested Laplace Approximation (INLA) approach was used to fit the spatial model. Lower cumulative rainfall, and higher accessibility were both associated with a higher RVFV seroprevalence. The spatial patterns of fitted RVFV seroprevalence pointed densely populated areas of western Senegal as being at higher risk of RVFV infection in small ruminants than rural or southeastern areas. Thus, because slaughtering infected animals and processing their fresh meat is an important RVFV transmission route for humans, more human populations might have been exposed to RVFV during the 2013–2014 outbreak than in previous outbreaks in Senegal.

## Author summary

An outbreak of Rift Valley fever (RVF)–a zoonotic mosquito-borne viral infection widespread in Africa, was reported in 2013–14 in Senegal. After its end in late 2014, its spatial distribution and drivers were assessed using a national serological survey in small ruminants, thus highlighting the high exposition of humans to RVF virus in urban areas.

## 1. Introduction

Rift Valley fever (RVF) is a zoonosis disease affecting wild and domestic ruminants, caused by an arbovirus that belongs to the genus *Phlebovirus* in the family *Phenuiviridae* of the order *Bunyavirales* [1]. The RVF virus (RVFV) is transmitted through (i) the bites of competent mosquito vectors, and/or (ii) contact with the body fluids of infected ruminants, the latter being the major route of transmission for human infection [2].

During RVF epidemics, the virus has been detected in many mosquito species belonging to at least 6 genera: *Aedes*, *Anopheles*, *Culex*, *Eretmapodites*, *Coquillettidia*, and *Mansonia* [3]. Among these, a few species of the *Culex* and *Aedes* genera appear to be the most suitable vectors for RVFV transmission [4].

The most frequently observed clinical signs during RVF outbreaks are mass abortions in pregnant ruminants and high mortality among young animals (mostly lambs) [4,5]. Sheep, goats, and cattle are susceptible to the disease leading to severe economic consequences [6]. Wild ruminants can act as a reservoir for the transmission and maintenance of the RVFV.

RVF outbreaks are triggered by heavy rainfall in dry areas, causing the proliferation of *Aedes* mosquitoes in temporary ponds. Neonate *Aedes* mosquitoes can get the RVFV either after biting infected ruminants, or by the transovarial route (quiescent infected *Aedes* eggs from the previous season). From these *primary foci* areas, animal mobility (transhumance, trade) can spread the RVFV to remote regions. It may be transmitted to animals, and humans, either by contact with infected blood and tissues, or by mosquitoes (*Culex*, *Aedes*, and *Anopheles*), thus causing *secondary foci*.

Since it was first isolated in Kenya in 1930 [7], RVFV was found in about 30 countries [3]. The first large RVF outbreak in Mauritania and Senegal was reported in 1987 [8]. Regional RVF surveillance implemented after this outbreak showed an heterogeneous RVFV circulation in space and time, mostly in southern Mauritania and neighbor areas. In October 1993, high RVFV transmission—associated with an increased abortion rate, were reported in small ruminants in the Ferlo region, north-central Senegal [9]. The RVFV was isolated from *Ae*. *vexans* mosquitoes in Barkedji (Ferlo), where no clinical cases were reported in local ruminants [10]. In 2003, small ruminant herds were affected by the disease in the Senegal River Delta and Valley [11], and RVFV transmission was observed in Barkedji. In 2013, several foci recorded ruminants from northern and central Senegal, and also, for the first time, in urban and peri-urban areas of Dakar and Thies. Moreover, six human cases were reported in the Thies region (Mbour district) [12]. Thus, there were evidences of an RVF epidemiology shift towards the South of the country.

Following this outbreak, a national serological survey was conducted in Senegal on the young ruminant population (6–18 months old) from November 2014 to January 2015, to identify the relative importance of two drivers of RVFV spread during this outbreak: environmental features (local spread), and trade-related ruminant mobility (remote spread). Young animals were chosen so to identify those who were infected during the 2013–2014 outbreaks.

## 2. Methods

### 2.1. Ethics statement

The research protocol for the study was approved by the International Vet school in Dakar (Senegal) and the Senegal veterinary services. Samples were taken from small ruminants with the farmers’ consent.

### 2.2. Study design for serological survey

**Fig 1:**
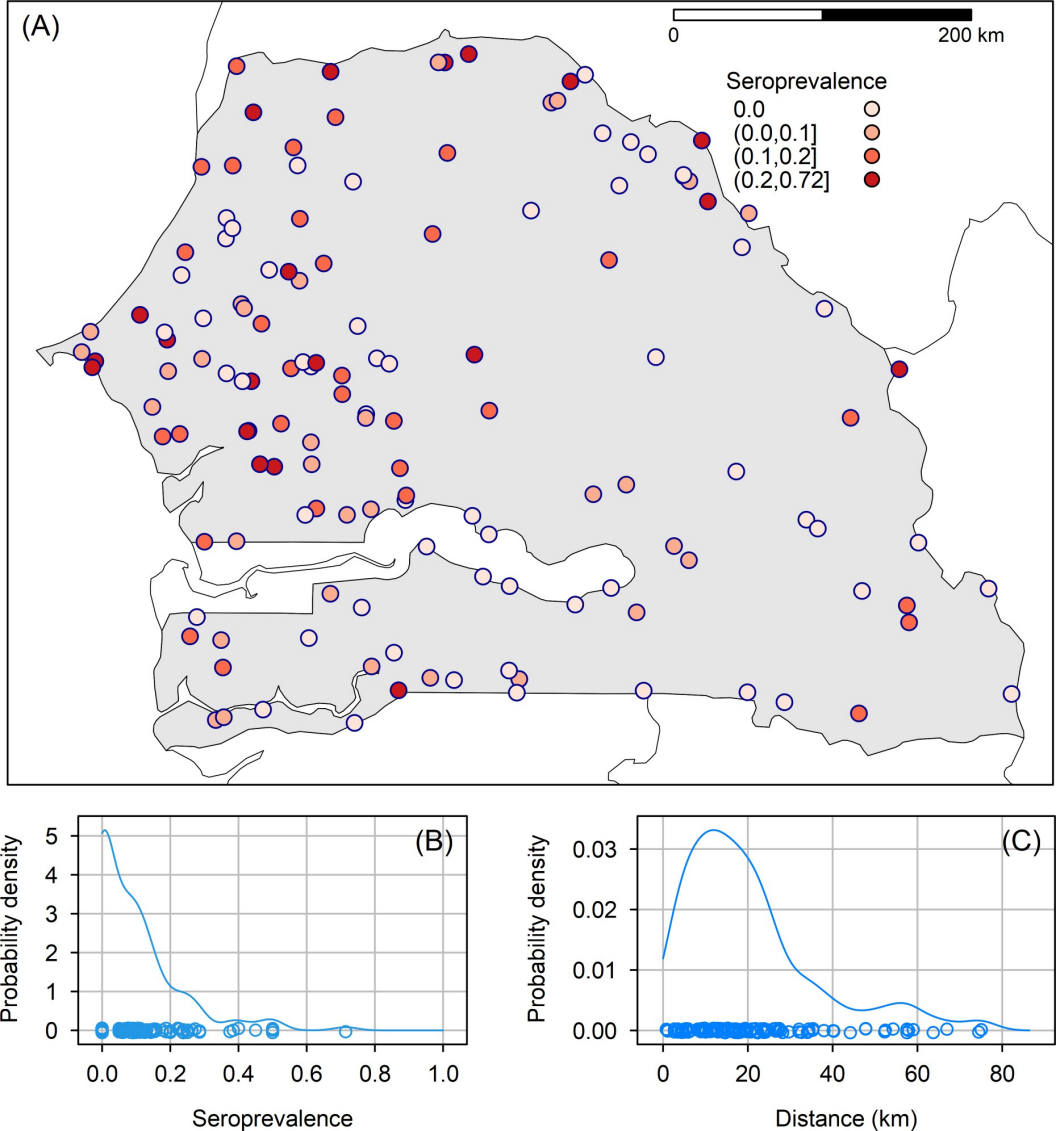
Observed RVFV seroprevalence in small ruminants in sampling locations after the rainy season 2014 in Senegal. (A) Spatial distribution; (B) Marginal distribution; (C) Distribution of smallest distances between sampling locations (primary source of the map: http://www.diva-gis.org/datadown).

A multi-stage sampling procedure was applied to estimate the number of villages and in each village, the minimum number of animals to be sampled. Settled villages in Senegal were the primary epidemiological units. A comprehensive and geo-referenced list of villages was provided by the Ecological Monitoring Center (*CSE*, Dakar). The list included *N* = 13,211 settled villages in 2014. Simple random sampling was designed based on the number of villages from this list, assuming a village-level prevalence (*p*_*v*_) of 10% as indicated by a national survey conducted after the 1987–88 RVF epidemics in Mauritania and Senegal [13]. Considering a confidence level of 95% and a desired precision *π* of 5%, using standard sample-size calculations, the number of villages to be sampled from across the country was n = 139. The randomly selected villages are presented in Fig 1.

Within these villages, *n*_*a*_ ruminants (cattle, sheep, or goats) between six- and eighteen-months old—i.e. born during the 2013–2014 RVF outbreak, were sampled. Thus, those animals testing positive for RVF (*m*_*a*_) were infected by the virus during the outbreak. Consecutively, the serorevalence, i.e., the proportion of positive animals can be considered as an incidence probability during the period of the outbreak.

The within-village sample size *n*_*a*_ was based on data reported after the 1987–88 RVF epidemic, when the estimated individual RVFV seroprevalence was 30% [8]. For this survey, we adopted a more conservative assumption of 20%, taking in account the sensitivity of the test, to estimate the within-village sample size to get at least one positive animal, i.e., *n*_*a*_ = 14.

### 2.3. Serological tests

Serums samples were tested for the presence of antibodies against RVFV with the RVF specific competitive Enzyme Linked Immuno Sorbent Assay (cELISA) based on the nucleoprotein N protein according to the manufacturer’s instructions. The specificity and sensitivity were estimated at 100% and 91–100% respectively [14] illustrating the high performances of the test used with reference to the gold standard technique (serum neutralization test) [15] that was previously used during the outbreak in Mauritania.

### 2.4. Predictor variables for RVFV transmission

Predictors were selected according to their known or plausible role in RVF epidemiology in Senegal [16] and split into two subsets: (i) drivers of local transmission involving mosquitoes and ruminants, (ii) drivers of RVFV spread through livestock movements (animals shedding RVFV).

#### 2.4.1. Drivers of local transmission of RVFV

Local RVFV transmission cycles are related to the joint presence of mosquito vectors and susceptible hosts. As information on vector abundance and activity could not be collected at the time of the national serological survey, several environmental factors related with vector ecology were considered instead:

- presence of watercourses (temporary or permanent) providing a suitable habitat for vector species;
- vegetation coverage providing suitable breeding and resting habitats for mosquitoes;
- rainfall patterns resulting in temporary ponds which trigger mosquito reproduction cycles; and
- night land surface temperature related to the development cycle of immature mosquitoes, as well as of RVFV within the infected mosquitoes.

These predictors were evaluated using composite data most often mixing field and remotely sensed data. Remote-sensing data were downloaded from publicly available website and scaled at the same resolution. For the presence of watercourses, a buffer of 10 km has been created around waterbodies and all location in the area was given value 1, 0 otherwise. For temporal series, like the vegetation coverage (estimated using normalized difference vegetation index—NDVI) and the surface temperature (estimated using the night land surface temperature—NLST), the minimum, the maximum, and the average values over the year 2014 were estimated. The list of predictors is provided in **Table 1**.

**Table 1:** Predictors of RVFV transmission and spread.

	Name	Description	Source	Role in RVFV epidemiology
Local RVFV Cycle	hydrodist	Distance from water sources	*Vmap0*	Surface water bodies are favorable locations for mosquito reproduction and contact between mosquitoes and ruminants at watering and resting time [17].
	Rfe	Cumulative rainfall intensity during the rainy season in Senegal (June-October)	*TAMSAT daily dataset*	Rainfall intensity and patterns essential in RVF epidemiology, for filling temporary ponds and starting mosquito reproduction [18]
	nevents	Number of dry spells during the rainy season (July to October), defined as time intervals > 10 days between ≥10-mm precipitations	*TAMSAT daily dataset*	
	NDVI	Availability of breeding and resting sites for mosquitoes	Normalized Difference Vegetation Indices	Higher values of NDVI reflect a higher vegetation coverage and potentially a higher availability of breeding and, especially, resting habitats for mosquitoes [19]
	minlst	Minimum Night land surface temperature	*USGS*	Mosquito activity and within-mosquito development of RVFV are related to temperature [20]
	Cattle, small ruminants	Density of cattle and small ruminants (log)	Gridded Livestock of the World, version 3.1	Principal animal hosts [21]
	Logdrat	Ratio small ruminants and cattle population	Gridded Livestock of the World, version 3.1	
RVF spread	logHmd	Human density (logarithm)	*Afripop*	The denser the human population, the stronger the demand for red meat—especially sheep in Senegal, thus resulting in more intense livestock trade. [22]
	logTrav2	Travel time needed to cross a one-km pixel (log). Accessibility defined as the inverse of travel time.	*JRC database*	Travel time is lower where human activities—including livestock trade, are more intense [23]
	Lcd2	Least cost distance between point and municipality centroids with direct connection to Mauritanian cases via incoming ruminant trade	Data collected by Veterinary Services on livestock mobility	RVFV could have been introduced during the 2013 epidemics in Mauritania, increasing the risk of exposure in the municipality directly connected [24]

#### 2.4.2. Drivers of RVFV remote spread

Drivers of RVFV remote spread were related to animal trade which in turn, depended on human density and demand for red meat, and accessibility, defined as the inverse of travel time needed to cross a given area [25]. Moreover, before the Tabaski religious celebration (Eïd el Kebir), the sheep demand is so strong that a greater percentage of sheep traded in Senegal are imported from southern Mauritania [26], thus increasing the risk of RVFV introduction. Based on these assumptions the selected remote-spread predictors were the human density, the travel times (i.e. the time needed to cross a one-km pixel) [23], and a constructed variable (lcd) estimating the least cost distance between survey locations and municipalities directly connected to Mauritanian foci areas. Data on livestock mobility were collected by the Senegalese Veterinary Services through the centralization and digitalization of “Laissez Passer Sanitaire”.

#### 2.4.3. Selection of predictors kept in the statistical models

Bivariate scatter plots of predictor variables were drawn to identify those with strong correlations (S3 Fig). The case being, we kept the one with the most straightforward link with the epidemiological process. Thus, NDVI was strongly correlated with the cumulative rainfall estimate rfe (estimated correlation coefficient $\hat{\rho }=0.73$); the log-density of human population (logHmd) was strongly correlated with the scaled density of cattle ($\hat{\rho }=0.66$), scaled density of small ruminants ($\hat{\rho }=0.65$), and least-cost distance to the nearest animal-introduction municipality ($\hat{\rho }=0.57$). The scaled density of small ruminants was also strongly correlated with the scaled density of cattle ($\hat{\rho }=0.87$). However, to keep track of the herd species composition, we introduced a new variable: the log-ratio of the densities of small ruminants and cattle: logdrat.

The predictor variables kept for statistical modelling were: the distance from water sources (hydrodist), the number of dry spells during the rainy season (nevents), the minimum night land surface temperature (minlst), the ratio of cattle to small ruminants (logdrat), and the log of travel time needed to cross a one-km pixel (logTrav2).

**Fig 2:**
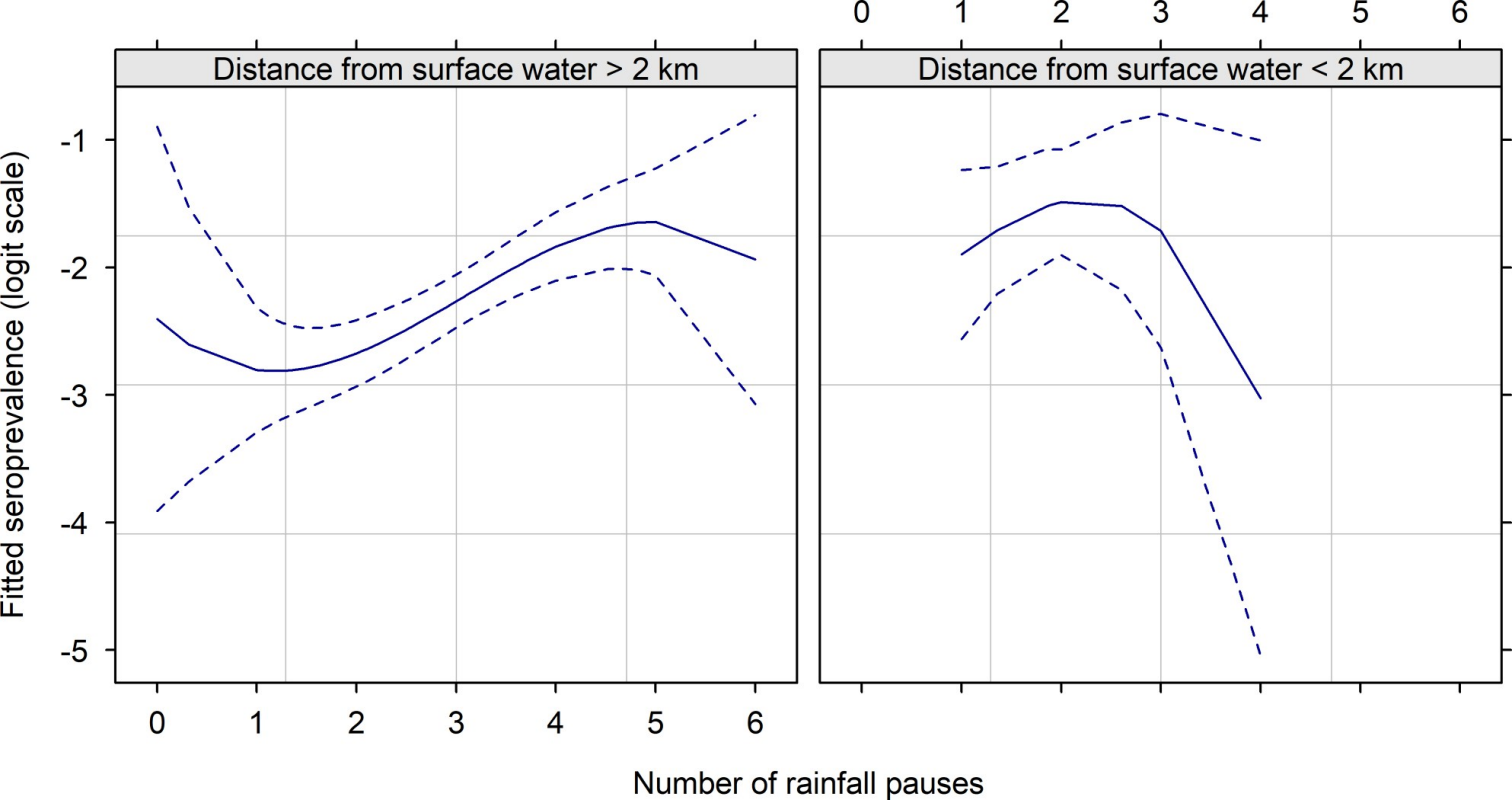
Logit of RVFV seroprevalence (solid line)—and 95% pointwise confidence interval (dashed lines), in small ruminants after the rainy season 2014 in Senegal, according to a spline function of the number of dry spells during the rainy season 2014, and conditionally on the distance to the nearest surface water.

The next step was to explore the intensity and shape of the link between a smoother of the predictor (i.e., a local mean), and the response on the logit scale to discard predictors unrelated with the response, considering possible non-linearities (hence the use of smoothers).A low number of rainfall events during the rainy season (Fig 2 on the right) was associated with a higher RVFV seroprevalence only when the sampled herds were located close to surface water bodies. On the other hand, a high number of dry spells during the rainy season when the sampled herds were located close to surface water bodies (Fig 2 on the left) was associated with a higher RVFV seroprevalence. Otherwise, this factor did not seem to affect the RVFV seroprevalence. We included these two main effects and their interactions in the statistical model.

**Fig 3:**
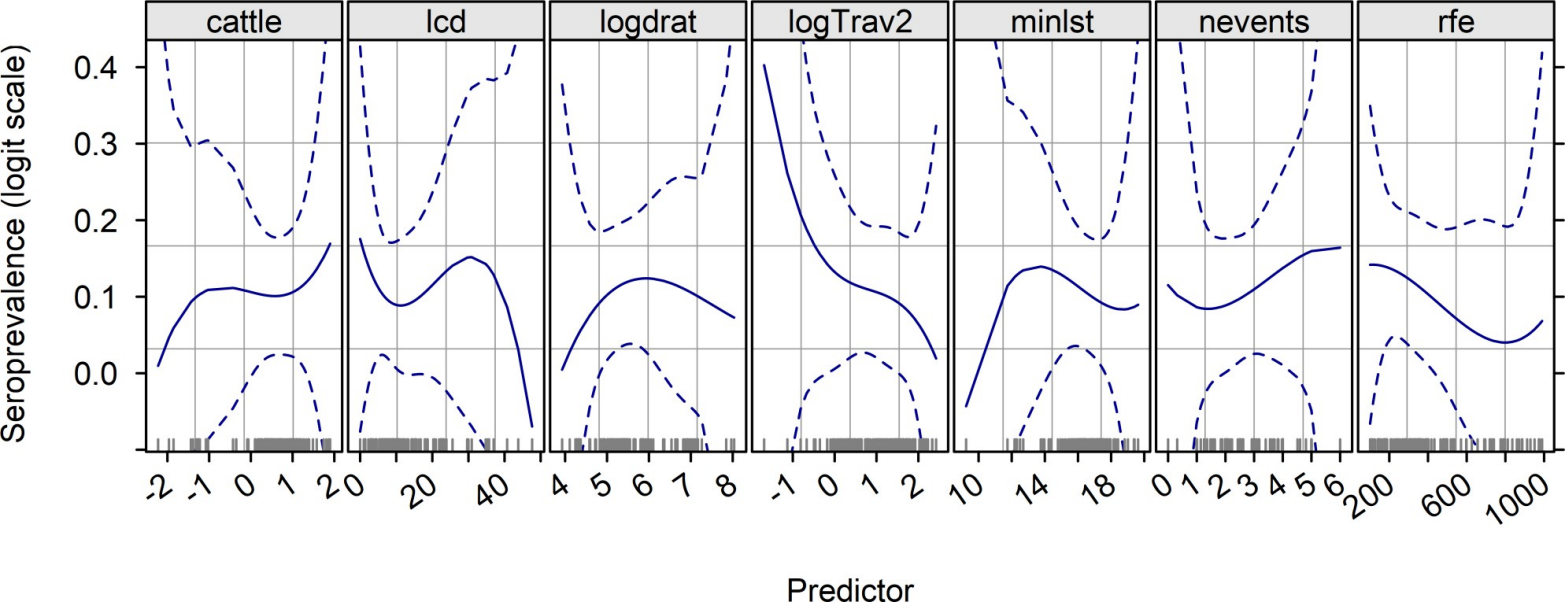
Logit of RVFV seroprevalence (solid line)—and 95% pointwise confidence interval (dashed lines), in small ruminants after the rainy season 2014 in Senegal, according to a spline function of its quantitative predictors. Codes for predictors: `cattle`log of scaled cattle density at sampling locations; `lcd`least-cost distance between the sampling locations and the centroid of the nearest municipality connected with southern Mauritania via incoming ruminant trade; `logdrat`log ratio of small ruminant to cattle densities at sampling locations; `logTrav2`log of travel time needed to cross a one-km pixel at sampling locations; `minlst`minimum night land surface temperature recorded at sampling locations during the rainy season 2014; `nevents`number of dry spells at sampling locations during the rainy season 2014; `rfe`cumulative rainfall at sampling locations in 2014, in Senegal.

The decrease of RVFV seroprevalence with higher rainfall (Fig 3) was in agreement with previous field observations that RVFV activity is higher in drier areas, probably because of the ecology of its vector mosquitoes. We included this item in the statistical model of RVF RVFV seroprevalence after transformation into rfe2 = (rfe—330) / 100, where 330 was the mean annual rainfall estimate for Senegal.

The effect of minimum night land surface temperature minlst was nonlinear (Fig 3, item minlst). However, because the number of samples associated with low minlst values was very small, we decided to discard this item from the multivariate model.

For the lowest values, an increase in the (log) ratio of small ruminant density over cattle density (i.e. higher proportion of small ruminants) was associated with higher RVFV seroprevalence (Fig 3, item logdrat). However, RVFV seroprevalence peaked around 6 (i.e., a small ruminant density *e*^6^ = 403)-fold higher than cattle density) and decreased afterward. We modeled this effect using the squared item, previously centered on the peak.

An increase of log-travel time was associated with a lower RVFV seroprevalence, in agreement with our expectations (**Fig 3,** item logTrav2). It was kept in the statistical model.

### 2.5. Statistical model

We used a beta-binomial logistic regression model of RVFV seroprevalence to account for possible over-dispersion of counts with respect to the binomial distribution, frequently met in epidemiology studies [27].

In the survey, many sampling sites were geographically close to each other (Fig 1A and 1C). The distance between sampling sites was less than 10 km in 28% of cases (Fig 1C). However, most predictors used in the model had a rather broad spatial resolution (1 to 10 km). While the beta-binomial distribution accounted for a general over-dispersion in the data, it did not explicitly account for a possible short-distance spatial correlation (e.g. related to micro-environmental features such as local mosquito abundance). Therefore, a spatial correlation term was added in the model, as a Matérn covariance function between sampling locations [28]. This function is controlled by two parameters: *κ*—the spatial scale parameter, and *ν*—the smoothing parameter, usually set to a fixed value. The spatial scale parameter *κ* identifies the distance at which the correlation becomes negligible.

To fit the model, an Integrated Nested Laplace Approximation (INLA) approach was used, combining analytical approximations and numerical integration (Markov Chain Monte Carlo). To account for the spatial auto-correlation in a computationally efficient way, the spatial scale parameter is estimated at the vertices of a spatial mesh (S4 Fig). The INLA procedure estimated posterior distribution of the parameters of interest [29]. The significance of the parameters was assessed by checking if their 95% CI overlapped or not the null value.

To identify the driving factors, we initially fitted the intercept-only model (with the spatial random effects), and progressively added predictors to minimize the deviance information criterion (DIC). The model, which minimized the DIC-value was considered as the best. In the model-selection process, we considered the addition of two-way interactions on the basis of their plausibility given our expertise on RVF epidemiology in this region. All statistical analyses were performed using R software and INLA-related analyses were implemented with the R package INLA [30].

## 3. Results

The village-level RVFV seroprevalence was high 59.4%, 95% confidence interval (CI) [50.6% - 68.2%]. Its spatial distribution showed a widespread RVFV infection in Senegal (Fig 1). However, the seroprevalence was highly heterogeneous in space, with many villages (40.6%), showing a null value mixed together with other villages with a high RVFV seroprevalence (Fig 1B).

**Fig 4:**
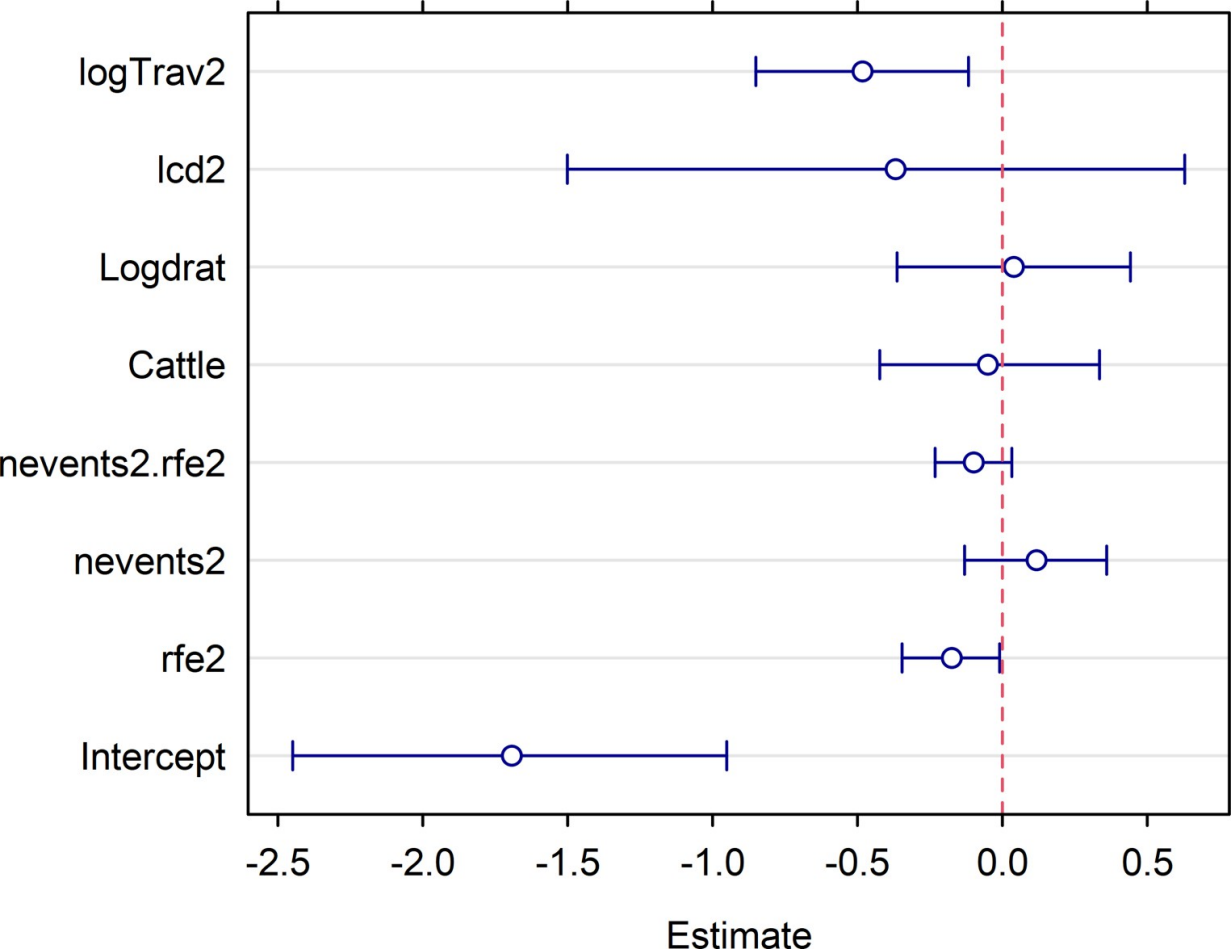
Posterior mean (circle) and 95% distribution interval (rod) of the predictor parameters for the DIC-best beta-binomial logistic regression model of RVFV seroprevalence in small ruminants after the 2013–2014 RVF outbreak in Senegal. The dashed, red vertical line shows the null hypothesis for the statistical test of these parameters (α = 0.05): it was rejected when this line fell out of the 95% distribution interval. Labels for the parameters code: ‘rfe2’ cumulative rainfall, ‘nevents2’: number of dry spells during the rainy season 2014, ‘nevents2:ref2’ interaction between these two covariates, ‘Cattle’ cattle density, ‘logdrat’ log ratio of small ruminant to cattle densities, ‘lcd2’ distance to the nearest municipality centroïd linked with southern Mauritania via ruminant incoming trade, ‘logTrav2’ log of travel time need to cross a one-km pixel.

The plot of fixed-effect parameters for the DIC-best spatial model of RVF seroprevalence (Fig 4) showed besides the intercept, travel time (logTrav2) and annual rainfall estimates were significantly lower than zero, i.e., more accessible and/or drier areas had a higher RVFV seroprevalence. The other predictors seemed to have more limited effects.

With AUC = 70%, the ROC curve for the DIC-best model of RVF seroprevalence (S5 Fig) showed this model had a fairly good predictive power. Thus, the identified predictors with a significant effect (both quantitatively and statistically) should point to meaningful features associated with RVF seroprevalence in small ruminants.

The range estimate in the spatial model of RVF seroprevalence was about 10 km, thus confirming our assumption of quick decrease in spatial correlation, probably related to micro-environmental features.

**Fig 5:**
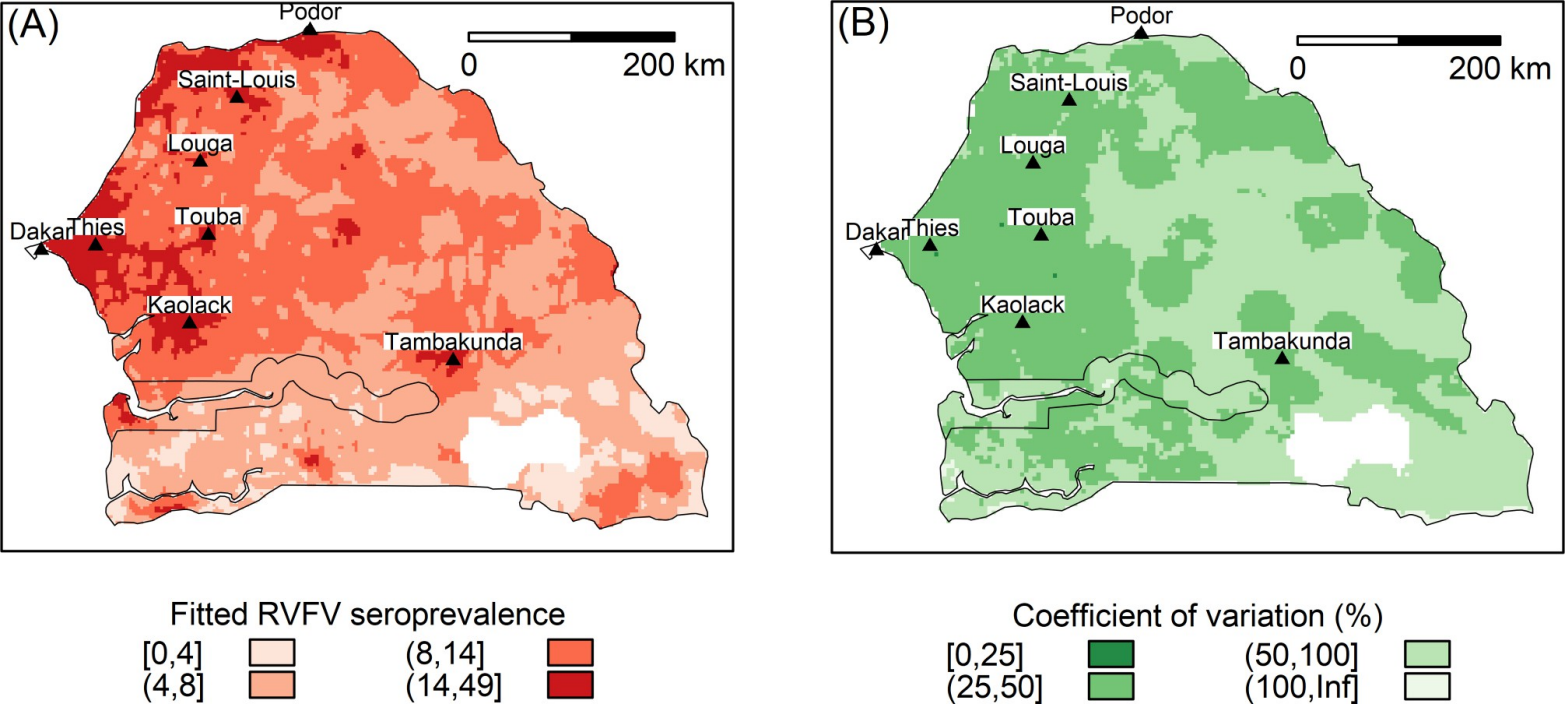
RVFV seroprevalence in small ruminants after the 2013–2014 RVF outbreak in Senegal (A) and its coefficient of variation (B) fitted by the DIC-best spatial beta-binomial logistic regression model (primary source of the map: http://www.diva-gis.org/datadown).

The distribution of fitted seroprevalence (Fig 5) showed highest values and best precisions along the coast and around densely-populated urban areas. The spatial distribution of the random effects (S6 Fig) showed random variation around the RVFV seroprevalence.

## 4. Discussion

The most important drivers of RVFV seroprevalence in small ruminants found in this study are in good agreement with our knowledge of RVF epidemiology in the region: (i) rainfall affects the ecology of RVF vector mosquitoes in particular the hatching of quiescent *Aedes* eggs, and (ii) livestock trade (with travel time as a proxy) favors remote spread of RVFV.

Contrary to previous observations [10], the analysis showed RVFV seroprevalence was higher around urban areas and on the coastal areas that are usually not involved in large transhumance movements [31]. This could indicate a change in anthropic factors driving the geographical spread of the infection. In 2013, RVF outbreaks were detected in September, during the rainy season, one month before the religious festivity of *Tabaski*, thus increasing the risk of outbreaks [8]. As a matter of fact, several hundred thousand of small ruminants (`~ 742,000 heads in 2014) were imported from Mauritania within a few weeks. Because Mauritania reported a major outbreak of RVF in 2012, the risk of introducing RVFV in Senegal via sheep trade was undoubtedly very high in 2012 and 2013. In addition, traded sheep are gathered in large animal marketplaces, thus providing multiple opportunities of direct contacts between infected and non-infected animals.

In terms of public health, an additional concern was the occurrence of RVF foci in urban environments. In Senegal, urban livestock farming represents an important source of income and food for urban dwellers [31]. In urban settings, animals live in close contact with other animals and humans, mostly in the owner’s backyard. Slaughtering infected animals and processing their fresh meat could increase the risk of transmission to humans. Moreover, livestock movements from countryside areas to provision urban consumer markets could exacerbate the risk. A surveillance system should be put in place to monitor the RVF disease situation in urban settings.

Spatial predictions using the DIC-best spatial beta-binomial logistic regression model of RVFV seroprevalence in small ruminants after 2013–2014 outbreak confirmed the observations made in 2013 [10]. Indeed, the spatial distribution was wider than usually reported during RVF outbreaks in Senegal [6], i.e. foci located in rural areas of northern Senegal (Delta and Valley of Senegal, fossil Valley of Ferlo)—showing primary-foci situation, surrounded by limited secondary spread. Conversely, in 2013 and 2014, RVFV was found in most regions of Senegal, except for the more humid areas on the South-Eastern borders. (Fig 5). However, the RVFV seroprevalence was very heterogeneous in space, and most of the areas exposed to a high risk of RVF were located in the densely populated regions of north-western Senegal. Therefore, these results suggest that during the rainy season in 2014, many people were exposed to the RVFV in urban and suburban areas of Dakar, Thiès, Kaolack, Tambacounda, Touba, Louga, and Saint-Louis (Fig 5).

In conclusion, the selection of predictors according to their known or plausible role in RVF epidemiology in Senegal included drivers of the local cycle RVFV transmission involving mosquitoes and ruminants, drivers of RVFV spread through livestock mobility. The data provide a good view of mobility patterns in the country; however, the information could not capture dynamics at a finer scale. Records of movements between several villages were missing in the data set. Only serological data was available for analysis, which could not provide any information about the time the virus appeared in an area–thus temporal spread could not be analyzed. Moreover, RVF specific seropositive animals could have been infected somewhere else before being traded in the villages. Despite limitations, results from our analysis suggest that in the risk analysis animal mobility and rainfall should be considered two of the main factors increasing the risk of RVFV cases. This information can be used to identify at-risk areas for active surveillance, to improve early detection, and/or targeted vaccination to prevent RVF outbreaks. Future work should aim to complete this information through retrospective studies on routine Veterinary Services data.

## Supporting information

S1 Fig Drivers of the local RVFV cycle considered in modelling the RVFV seroprevalence in small ruminants after the rainy season 2014 in Senegal. Senegal (A) Two-km buffered hydrographic network; (B) Rainfall pauses during the rainy season 2014; (C) Maximum NDVI from June to October 2014, (D) Minimum weekly night land surface temperature from June to October 2014 (primary source of the map: *http://www.diva-gis.org/datadown*).
(DOCX)

S2 Fig Drivers of RVFV spread considered in modelling RVFV seroprevalence in small ruminants after the rainy season 2014 in Senegal. (A) Cattle density; (B) Small ruminant density; (C) Travel time between cities > 50,000 inhab (log scale); (D) Shortest least-cost distance between a given pixel and the centroïd of municipalities where livestock was introduced from RVF high-risk areas (primary source of the map: *http://www.diva-gis.org/datadown*).
(DOCX)

S3 Fig Correlation of predictors of RVFV seroprevalence in small ruminants in Senegal after the rainy season 2014. The name of predictors of RVFV seroprevalence are drawn on the diagonal together with their probability density. The upper-diagonal panels show the bivariate scatterplots as well as a loess-smoothing line. The bivariate linear correlation coefficients are shown in the lower-diagonal panels. ‘nevents’ number of dry spells during the rainy season 2014, ‘rfe’ cumulative rainfall during the rainy season 2014, ‘ndvi’ fifth centile of a series of 8-day composite remotely sensed records of maximum normalized vegetation index during the rainy season 2014, ‘minlst’ of a series of 8-day composite remotely sensed records of minimum night land surface temperature during the rainy season 2014, ‘logHmd’ log of human density at the municipality level, ‘cattle’ cattle density at the municipality level, ‘shoats’ small ruminant density at the municipality level, ‘logdrat’ log ratio of small ruminant to cattle densities at the municipality level, ‘logTrav2’ log travel time needed to cross a one-km pixel, ‘lcd’ least-cost distance from the sampling location to the centroid of the nearest municipality linked to southern Mauritania via incoming ruminant trade.
(DOCX)

S4 Fig Mesh used to estimate the Gaussian random field, and consequently the Matérn correlation function of the spatial beta-binomial logistic regression model of RVFV seroprevalence in small ruminants after the rainy season 2014 in Senegal. (DOCX)

S5 Fig ROC curve for the DIC-best spatial beta-binomial model of RVFV seroprevalence in small ruminants after the rainy season 2014 in Senegal. (DOCX)

S6 Fig Estimated Gaussian random fields in the DIC-best spatial beta-binomial logistic regression model of RVFV seroprevalence in small ruminants after the rainy season 2014, Senegal (A) Mean; (B) Standard error (primary source of the map: *http://www.diva-gis.org/datadown*).
(DOCX)

S1 Table Estimated hyperparameters for the random effects included in the DIC-best spatial beta-binomial logistic regression model of RVFV seroprevalence in small ruminants after 2013 epidemics in Senegal. (XLSX)
