# Supplementary material for: Identification of drivers of Rift Valley fever after the 2013–14 outbreak in Senegal using serological data in small ruminants
Source: PLoS Negl Trop Dis. 2022 Feb 2;16(2):e0010024. doi: 10.1371/journal.pntd.0010024 (PMC8843136; doi:10.1371/journal.pntd.0010024)
Supplement: S1 Fig — Senegal (A) Two-km buffered hydrographic network; (B) Rainfall pauses during the rainy season 2014; (C) Maximum NDVI from June to October 2014, (D) Minimum weekly night land surface temperature from June to October 2014 (primary source of the map: http://www.diva-gis.org/datadown). (DOCX) [file pntd.0010024.s001.docx]

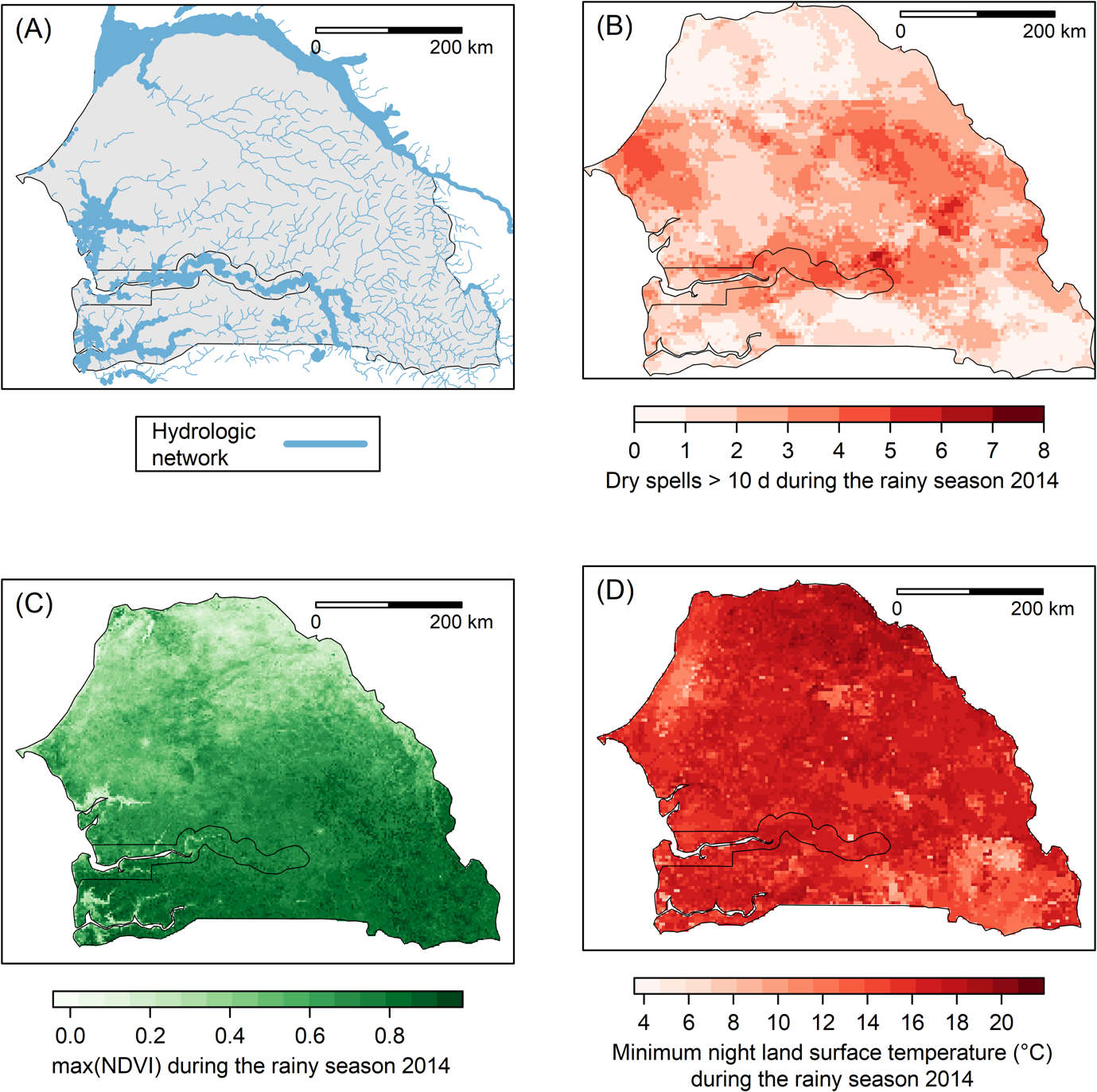


**S1 Fig**: Drivers of the local RVFV cycle used to model RVFV seroprevalence in small ruminants after the rainy season of 2014 in Senegal (A) Two-km buffered hydrographic network; (B) Rainfall pauses during the rainy season 2014; (C) Maximum NDVI from June to October 2014, (D) Minimum weekly night land surface temperature from June to October 2014.
