# Supplementary material for: Identification of drivers of Rift Valley fever after the 2013–14 outbreak in Senegal using serological data in small ruminants
Source: PLoS Negl Trop Dis. 2022 Feb 2;16(2):e0010024. doi: 10.1371/journal.pntd.0010024 (PMC8843136; doi:10.1371/journal.pntd.0010024)
Supplement: S2 Fig — (A) Cattle density; (B) Small ruminant density; (C) Travel time between cities > 50,000 inhab (log scale); (D) Shortest least-cost distance between a given pixel and the centroïd of municipalities where livestock was introduced from RVF high-risk areas (primary source of the map: http://www.diva-gis.org/datadown). (DOCX) [file pntd.0010024.s002.docx]

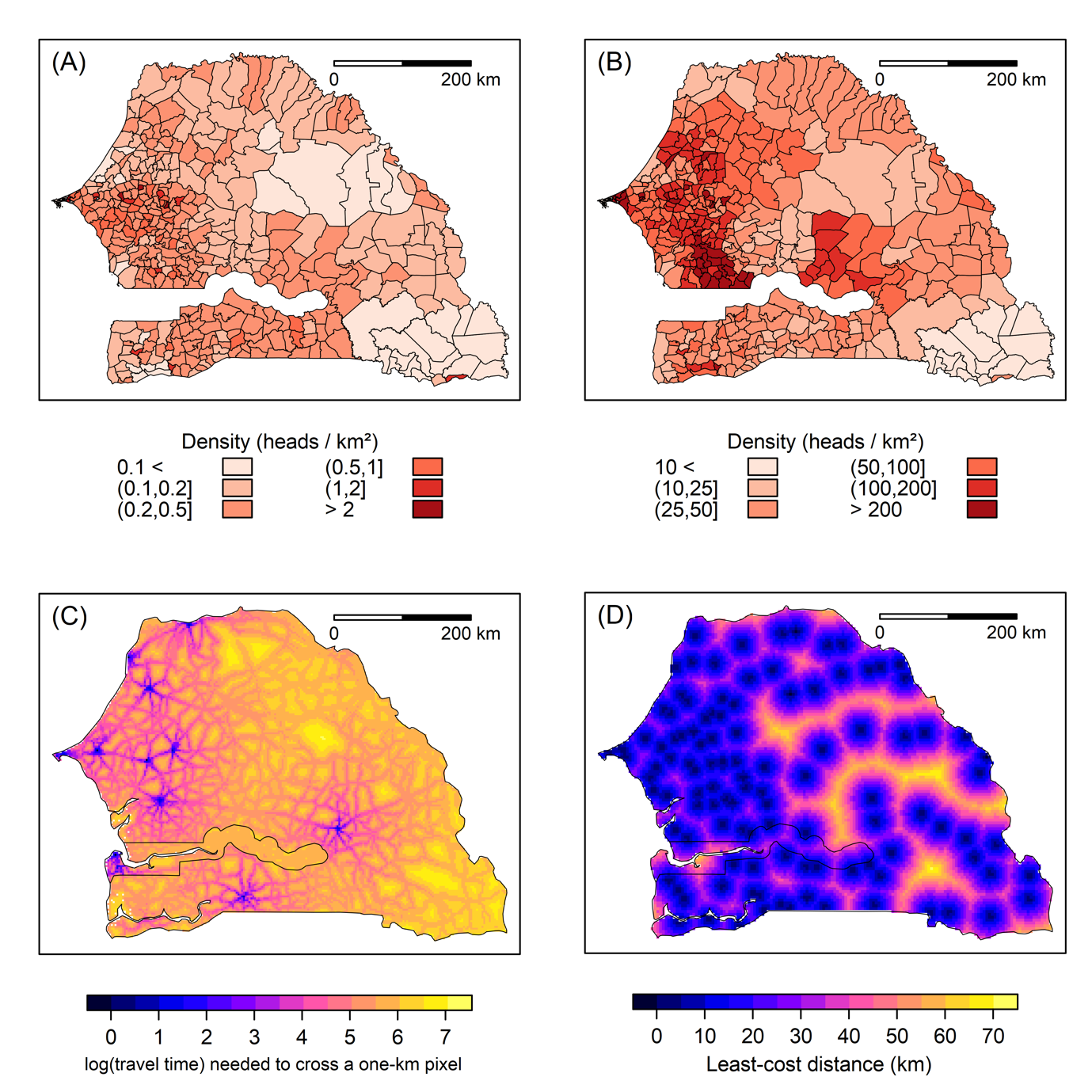


**S2 Fig:** Drivers of RVFV spread used to model the RVVF seroprevalence in small ruminants after the rainy season 2014 in Senegal. (A) Cattle density; (B) Small ruminant density; (C) Travel time between cities > 50,000 inhab (log scale); (D) Shortest least-cost distance between a given pixel and the centroïd of municipalities where livestock was introduced from RVF high-risk areas.
