# Supplementary material for: Identification of drivers of Rift Valley fever after the 2013–14 outbreak in Senegal using serological data in small ruminants
Source: PLoS Negl Trop Dis. 2022 Feb 2;16(2):e0010024. doi: 10.1371/journal.pntd.0010024 (PMC8843136; doi:10.1371/journal.pntd.0010024)
Supplement: S3 Fig — The name of predictors of RVFV seroprevalence are drawn on the diagonal together with their probability density. The upper-diagonal panels show the bivariate scatterplots as well as a loess-smoothing line. The bivariate linear correlation coefficients are shown in the lower-diagonal panels. ‘nevents’ number of dry spells during the rainy season 2014, ‘rfe’ cumulative rainfall during the rainy season 2014, ‘ndvi’ fifth centile of a series of 8-day composite remotely sensed records of maximum normalized vegetation index during the rainy season 2014, ‘minlst’ of a series of 8-day composite remotely sensed records of minimum night land surface temperature during the rainy season 2014, ‘logHmd’ log of human density at the municipality level, ‘cattle’ cattle density at the municipality level, ‘shoats’ small ruminant density at the municipality level, ‘logdrat’ log ratio of small ruminant to cattle densities at the municipality level, ‘logTrav2’ log travel time needed to cross a one-km pixel, ‘lcd’ least-cost distance from the sampling location to the centroid of the nearest municipality linked to southern Mauritania via incoming ruminant trade. (DOCX) [file pntd.0010024.s003.docx]

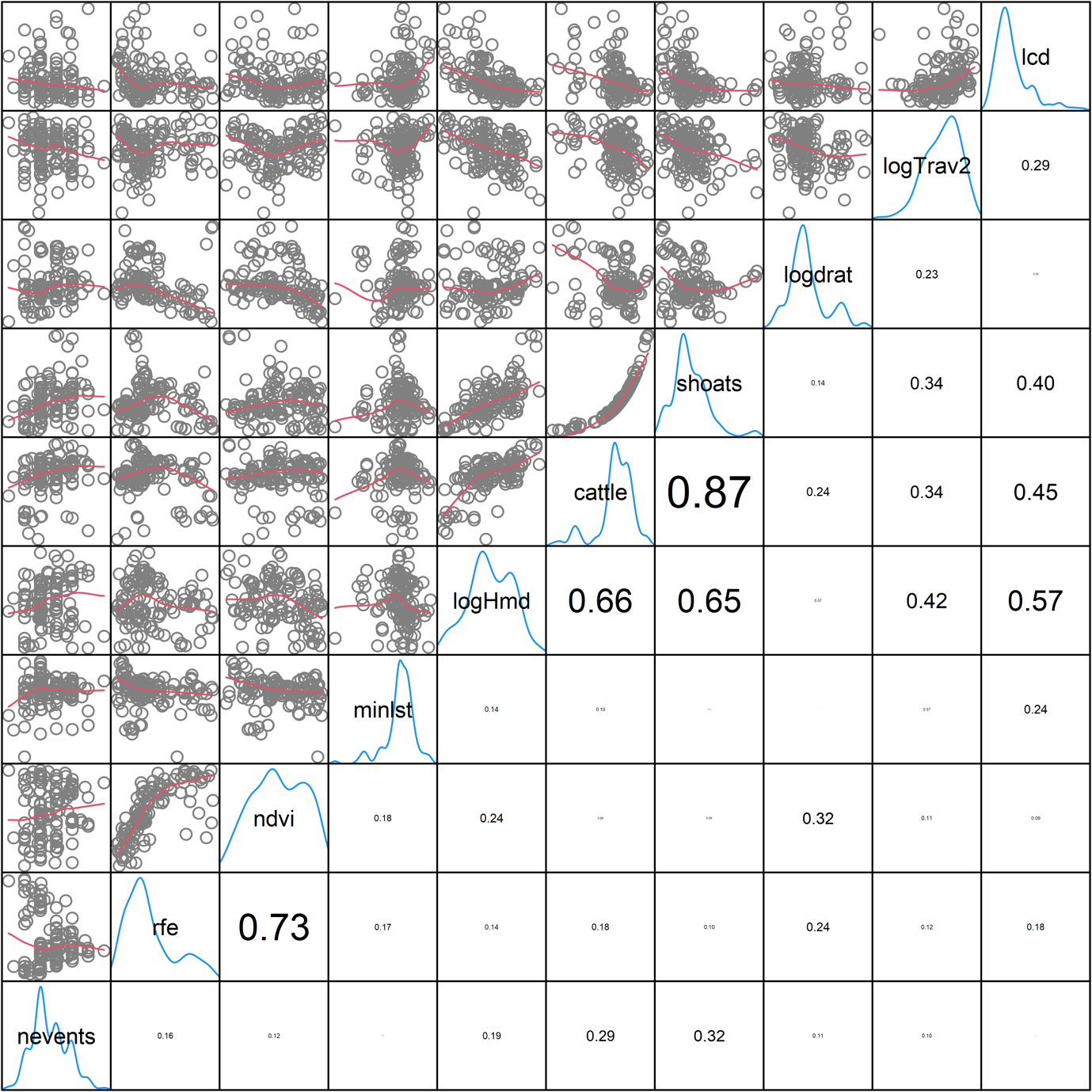


**S3 Fig**: Correlation of predictors of RVFV seroprevalence in small ruminants in Senegal after the rainy season 2014. The name of predictors of RVFV seroprevalence are drawn on the diagonal together with their probability density. The upper-diagonal panels show the bivariate scatterplots as well as a loess-smoothing line . The bivariate linear correlation coefficients are shown in the lower-diagonal panels. ‘nevents’ number of dry spells during the rainy season 2014, ‘rfe’ cumulative rainfall during the rainy season 2014, ‘ndvi’ fifth centile of a series of 8-day composite remotely sensed records of maximum normalized vegetation index during the rainy season 2014, ‘minlst’ of a series of 8-day composite remotely sensed records of minimum night land surface temperature during the rainy season 2014, ‘logHmd’ log of human density at the municipality level, ‘cattle’ cattle density at the municipality level, ’shoats’ small ruminant density at the municipality level, ‘logdrat’ log ratio of small ruminant to cattle densities at the municipality level, ‘logTrav2’ log travel time needed to cross a one-km pixel, ‘lcd’ least-cost distance from the sampling location to the centroid of the nearest municipality linked to southern Mauritania via incoming ruminant trade.
