# Supplementary material for: Identification of drivers of Rift Valley fever after the 2013–14 outbreak in Senegal using serological data in small ruminants
Source: PLoS Negl Trop Dis. 2022 Feb 2;16(2):e0010024. doi: 10.1371/journal.pntd.0010024 (PMC8843136; doi:10.1371/journal.pntd.0010024)
Supplement: S4 Fig — (DOCX) [file pntd.0010024.s004.docx]

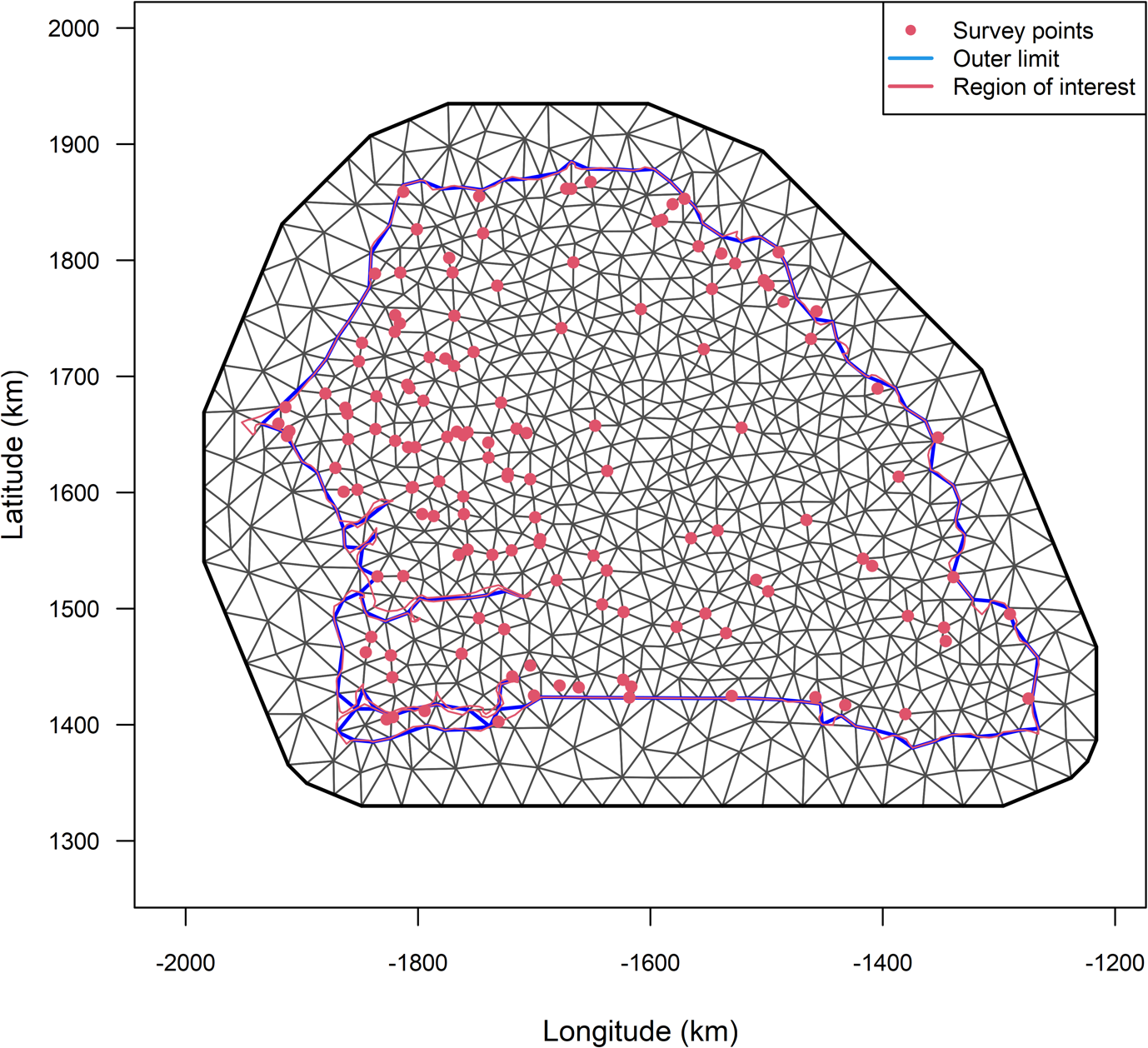


**S4 Fig**: Mesh used to estimate the Gaussian random field, and consequently the Matérn correlation function of the spatial beta-binomial logistic regression model of RVFV seroprevalence in small ruminants after the rainy season 2014 in Senegal.
