# Supplementary material for: Identification of drivers of Rift Valley fever after the 2013–14 outbreak in Senegal using serological data in small ruminants
Source: PLoS Negl Trop Dis. 2022 Feb 2;16(2):e0010024. doi: 10.1371/journal.pntd.0010024 (PMC8843136; doi:10.1371/journal.pntd.0010024)
Supplement: S6 Fig — Estimated Gaussian random fields in the DIC-best spatial beta-binomial logistic regression model of RVFV seroprevalence in small ruminants after the rainy season 2014, Senegal (A) Mean; (B) Standard error (primary source of the map: http://www.diva-gis.org/datadown). (DOCX) [file pntd.0010024.s006.docx]

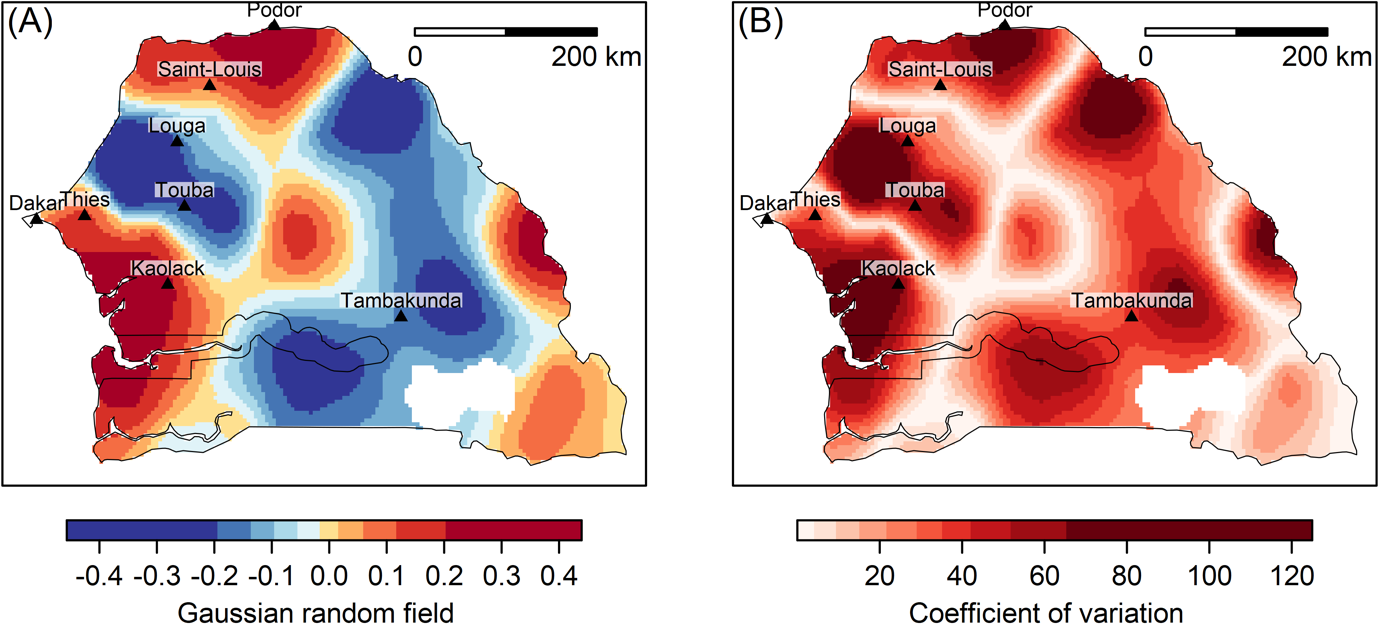


**S6 Fig:** Estimated Gaussian random fields in the DIC-best spatial beta-binomial logistic regression model of RVFV seroprevalence in small ruminants after the rainy season 2014, Senegal. (A) Mean; (B) Standard error
